# Behavioral Study of Elastomeric O-Rings Built into Coaxial Sealing Systems

**DOI:** 10.3390/polym17091275

**Published:** 2025-05-07

**Authors:** Andrea Deaconescu, Tudor Deaconescu

**Affiliations:** Department of Industrial Engineering and Management, Transilvania University of Brasov, 500036 Brasov, Romania; deacon@unitbv.ro

**Keywords:** O-rings, coaxial sealing systems, elastomers

## Abstract

Coaxial sealing systems are increasingly used in the construction of hydraulic cylinders. In addition to the seal that ensures the actual packing of the entire system, the O-ring plays an important role in the functioning of the hydraulic subassembly. In order to understand the sealing phenomenon of coaxial systems, a physical and mathematical model of the contact between the O-ring and its contacting surfaces is required. Within this context, this paper presents a calculation method of the pressures generated in the contact areas of the O-ring with its adjacent surfaces, as well as of the widths of the contact areas. The input quantities for these calculations were certain material characteristics (hardness, elasticity modulus, and Poisson’s coefficient) of the sealed-off fluid pressure and the specific radial deformation, which is a characteristic that describes the mounting of the O-ring in its groove. This article concludes with recommendations for the mounting of the O-ring and the required characteristics of the used materials.

## 1. Introduction

Coaxial sealing systems are specialized sealing solutions often used in various industrial applications to prevent the leakage of fluids. These systems typically consist of concentric sealing elements that are arranged in a way to provide effective sealing under different operational conditions. This design helps to distribute stresses evenly and can accommodate thermal expansion and contraction. The concentric design allows for better control over the sealing pressure and reduces the risk of leaks, even in high-pressure or high-temperature environments [1].

The main fields of applicability of these sealing systems are aerospace and automotive (engine seals and fuel systems), sectors like oil and gas, chemical processing, and manufacturing (used in cylinders and valves to maintain pressure and prevent the escape of hydraulic fluids or gases).

Coaxial sealing systems can be designed for both dynamic (moving parts) and static (stationary parts) applications, making them versatile for various types of machinery and equipment. These systems can be tailored to fit specific applications, including those that require resistance to extreme temperatures, chemicals, or high pressures. While coaxial sealing systems can offer an enhanced performance, they may also come with a higher initial cost compared to simpler sealing solutions [2].

**Figure 1:**
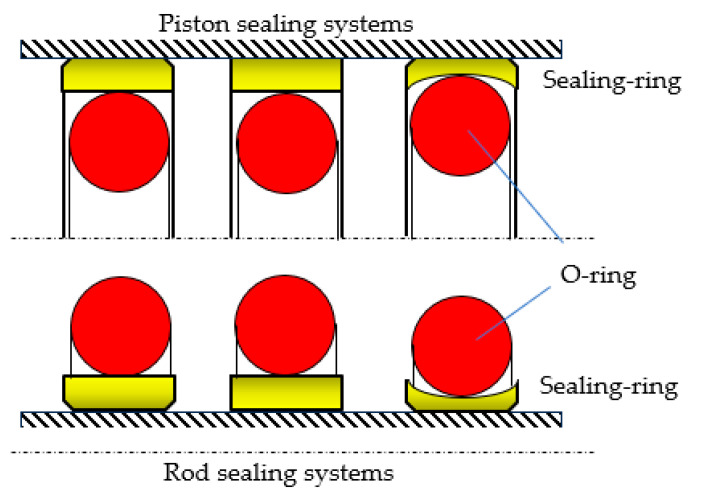
Components of a coaxial sealing system.

A coaxial sealing system consists of two elements: (i) a ring (seal) made from a material with excellent friction properties that comes into contact with the sealed-off surface, and (ii) an O-ring that ensures the tensioning of the entire assembly (Figure 1) [3]. An efficient sealing is obtained by applying a radial compression force onto the seal by the O-ring and the pressure of the sealed-off fluid. In order to generate the radial pressure, the O-ring is placed in its groove in a pre-compressed state, with a specific initial radial deformation of 10–25%. Once the fluid pressure in the hydraulic motor increases, the O-ring deforms additionally, which leads to the generation of an even greater radial pressure [4,5].

While the O-ring plays a significant role in the sealing process, only a few studies have been published about its behavior within coaxial sealing systems. One such study, authored by Wang et al. [6], analyzes the behavior of coaxial sealings of GS (gland seal) type used in hydro-pneumatic suspensions. The research results provide a theoretical basis for the design of GS sealing of hydro-pneumatic springs and the effective improvement of the life and reliability of related equipment. The contact pressures generated by the O-rings on the surfaces of the sealing system were studied in ref. [7,8]. In ref. [7], a physical–mathematical model of the contact between the sealing O-ring and the sealing surfaces is discussed, and the maximum values of the contact pressures between the sealing ring and the cylindrical sealing surfaces are calculated. Aissaoui et al. present in ref. [8] the contact stress profiles and the peak contact stresses generated by O-rings onto the contact surfaces. An important research direction in the field of hydraulic systems sealing concerns the materials these systems are made from. The majority of seals are made from polymers, in particular elastomers, plastomers, or thermoplastic elastomers. The choice of material depends on the specific operating conditions, including compatibility with the sealed-off fluid, its pressure, temperature, etc. A long lifetime for sealing systems calls for in-depth knowledge of the used materials. Selecting the most adequate materials for a specific application is crucial for ensuring compatibility with the sealed-off fluids and the working environment. The most frequently used material for seals is polytetrafluoroethylene (virgin or filled PTFE), while O-rings are made from various types of elastomers: nitrile rubber (NBR), hydrogenated nitrile rubber (HNBR), fluorocarbon (FKM), fluorosilicone (FVMQ), ethylene propylene diene monomer rubber (EPDM), and silicone rubber (Q) [4].

Research related to the materials used for hydraulic sealing systems dates back more than 90 years [9]. In ref. [10], B.S. Nau summarizes the knowledge to date of the factors that influence the performance of hydraulic seals made from polymers, and in ref. [11], the same author addresses rubber seals.

Studies concerning the use of plastomers for sealing systems can be found in numerous articles, like in refs. [12,13,14,15,16]. These papers highlight phenomena like abrasive wear and adhesion of PTFE seals, as well as the effects of fatigue caused by the high temperatures of the working fluid. Virgin PTFE has limited applications due to its low wear strength. Such obstacles are overcome by inserting additives into the PTFE matrix, yielding PTFE-based composites more suitable for hydraulic actuation systems. Additives used together with PTFE are glass fibers, carbon fibers, graphite, copper particles, molybdenum disulfide, and mixes thereof [17]. Additives like bronze, glass fibers, Mo_2_S, or carbon present in the PTFE composition have different influences on the behavior of seals. Thus, for example, bronze improves wear strength at environmental temperatures, but has the opposite effect at high temperatures.

Over the last year, numerous research works have studied the behavior of O-rings made from various materials during the sealing process. In ref. [18], the authors studied the behavior of O-rings made from EPDM under extremely high and low temperature conditions. The conclusions show that the stress concentration phenomenon of the EPDM rubber O-ring is lighter under high temperature conditions, but the sealing performance decreases. At low temperatures, the stress concentration is significant, but the sealing performance is better. Kömmling et al. present in ref. [19] a method for determining a specific end-of-lifetime criterion for O-ring seals. Determining a suitable and reliable end-of-lifetime criterion for O-ring seals is an important issue for long-term seal applications. 

Müller et al. studied in ref. [20] the compatibility of elastomer-based sealing O-rings with methanol, ethanol, and hydrotreated vegetable oil for fueling internal combustion engines. Among the different elastomer O-rings tested, the best performance in terms of material compatibility and dependence on the fuels was exhibited by the FVMQ (fluorosilicone elastomer) type O-rings.

Huang et al. developed in ref. [21] a model for predicting the leakage rate that affects the sealing performance of O-rings made from ethylene propylene diene monomer rubber (EPDM). The model was proven to be an accurate predictor of the onset of leakage and was validated by a vacuum test.

The numerous and intensive endeavors leading up to the current state of development of coaxial sealing systems underpin the fact that these systems are a significant aspect of future sealing technology. As industry evolves and is faced with new challenges, the development and optimization of coaxial sealing systems remain critical areas of attention. In this regard, this paper proposes to analyze a less explored area, namely the operational behavior of O-rings made from polymeric materials. The analyzed O-rings are used for the sealing of hydraulic cylinders that carry out linear motions. This paper includes the following sections: after the introductory chapter, the second part of the paper describes the structure of a coaxial sealing system and introduces the main materials used at present in the manufacturing of O-rings. Further on, Section 3 presents the behavior of the O-ring during mounting and under pressure. The pressure distributions in the contact areas of the O-ring with the adjacent walls are described, and hypotheses are developed leading to the relationships used to calculate the contact area widths. Based on the obtained relationship, graphs are presented describing the dependencies of contact area widths and contact pressure on the specific radial deformation, on the hardness of the O-ring material, and on the pressure of the working fluid. The fourth section of the paper contains the discussion of the obtained results, and the fifth section presents the main conclusions of the conducted study.

## 2. Materials

**Figure 2:**
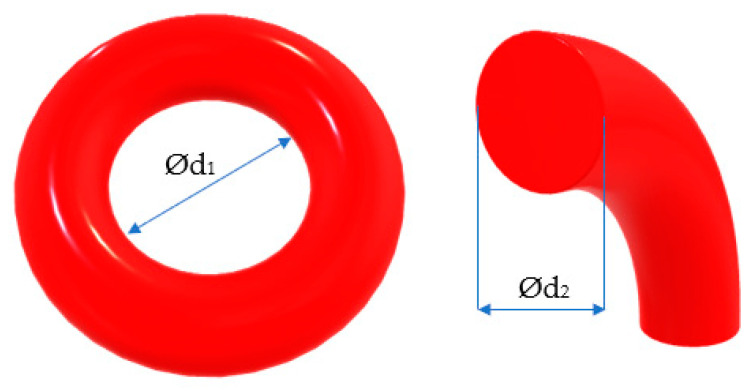
Characteristic dimensions of an O-ring.

The O-ring included by coaxial sealing systems is described by two dimensional characteristics (Figure 2): the interior diameter *d*_1_ and the diameter of the cross section *d*_2_.

O-rings are made mainly from rubber materials, as these are cost-effective and hold their shape well, making them ideal for use in sealing applications. The most frequently used materials are NBR (nitrile butadiene rubber), EPDM (ethylene propylene diene monomer rubber), VITON (fluoroelastomer synthetic rubber), VMQ (silicone rubber), and CR (neoprene/chloroprene rubber). These are used in a wide range of engineering applications (static and dynamic seals, belts, support inserts, vibration insulators, etc.). Table 1 shows a number of the polymeric materials used for O-rings, together with their characteristics [22,23].

In most industrial applications, the polymeric composites O-rings are made from have a hardness of 70–80 Shore A. In dynamic applications, a greater hardness (90 Shore A) can compromise the sealing by leaking a few drops of liquid, while a smaller hardness (50 Shore A) can cause abrasion, wear, or extrusion [23]. Strictly in the case of coaxial systems, the O-ring ensures static sealing that will be analyzed further on.

## 3. Method and Results

**Figure 3:**
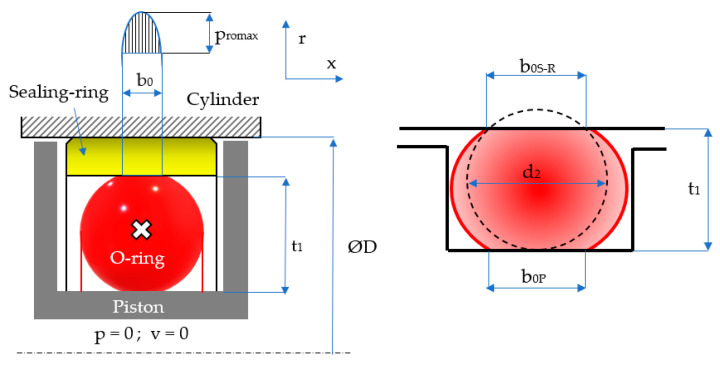
Pre-compressed O-ring placed in its groove.

**Figure 4:**
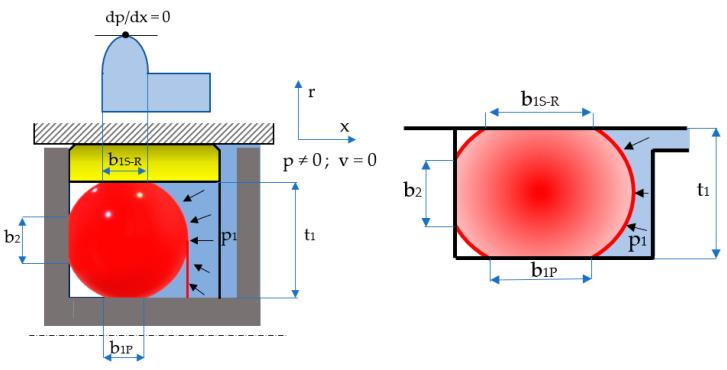
Applying pressure to the sealed-off fluid.

Sealing the pistons of hydraulic cylinders by means of coaxial sealing systems is achieved through a number of steps. Initially, the O-ring is placed in its groove in a pre-compressed state while fluid pressure is zero (Figure 3). Once pressure is applied, the O-ring deforms additionally, proportionally to the magnitude of the pressure (Figure 4).

The notations in the figures above are: *b*_0*S-R*_ and *b*_0*P*_ are the widths of the contact areas between the O-ring and the adjacent walls in the absence of working fluid pressure (index *S*-*R* refers to the contact of the O-ring with the sealing ring, and index *P* is attributed to the contact area with the piston walls); and *b*_1*S-R*_ and *b*_1*P*_ are the widths of the contact areas in the presence of working fluid pressure. The simplified representation on the right-hand side of Figure 4 highlights the behavior of the O-ring similar to a static sealing system, as its only role is to generate the radial tensioning of the sealing ring. This figure also shows that the O-ring is closed in on three sides, thus preventing extrusion.

### 3.1. Behavior of the O-Ring in the Absence of the Fluid Pressure to Be Sealed-Off

Upon being mounted in its groove, the O-ring modifies its geometry due to deformation in two directions, an axial and a radial one. The specific radial deformation (*ε_r_*_0_) is dependent on the initial diameter of the O-ring cross-section (*d*_2_) and on the depth of the groove (*t*_1_); this dependency is described by Equation (1):

(1) $$\varepsilon_{ro}=\frac{d_{2}-t_{1}}{d_{2}}$$

Depending on the specific deformation, the shape of the O-ring cross-section is modified by the increase in the contact area width *b*_0_. The relationships calculating these quantities have been presented in a number of papers. Thus, for example, Lindley proposes in ref. [24,25] a formula for calculating the width of the contact area:

(2) $$b_{0}=d_{2}\cdot \sqrt{\frac{6}{\pi }\cdot \left(1.25\cdot \varepsilon_{ro}^{1.5}+50\cdot \varepsilon_{ro}^{6}\right)}$$

Karaszkiewicz also proposes such a formula in ref. [26]:

(3) $$b_{0}=d_{2}\cdot \left(2\cdot \varepsilon +0.13\right)\text{, applicable for 0.07 ≤}\;\varepsilon \;\text{≤ 0.25}$$

In order to determine the magnitude of the contact area, the following relationships can be deduced using the notations in Figure 3:

(4) $$\bar{OB}=\frac{d_{2}}{2}\cdot \left(1+\varepsilon_{xo}\right)$$

where *ε_x_*_0_ is the specific axial deformation, and:

(5) $$\frac{b_{0}}{2}=\sqrt{\frac{d_{2}^{2}}{4}\cdot {\left(1+\varepsilon_{xo}\right)}^{2}-\frac{d_{2}^{2}}{4}\cdot {\left(1-\varepsilon_{ro}\right)}^{2}}$$

In the absence of fluid pressure, the relationship describing the dependency between the specific radial and axial deformations is:

(6) $$\varepsilon_{xo}=-\nu \cdot \varepsilon_{ro}$$

where *ν* is Poisson’s coefficient for the O-ring’s material. For the analyzed materials (Table 1), a value of *ν* = 0.496 is adopted in calculations. The value of Poisson’s coefficient in the case of elastomers (*ν* ≈ 0.5) means that they deform without changing their volume (perfectly incompressible isotropic materials).

Upon introducing Equation (6) into (5), there results:

(7) $$b_{0}=d_{2}\cdot \sqrt{\varepsilon_{ro}^{2}\cdot \left(\nu^{2}-1\right)+2\cdot \varepsilon_{ro}\cdot \left(1-\nu \right)}$$

**Figure 5:**
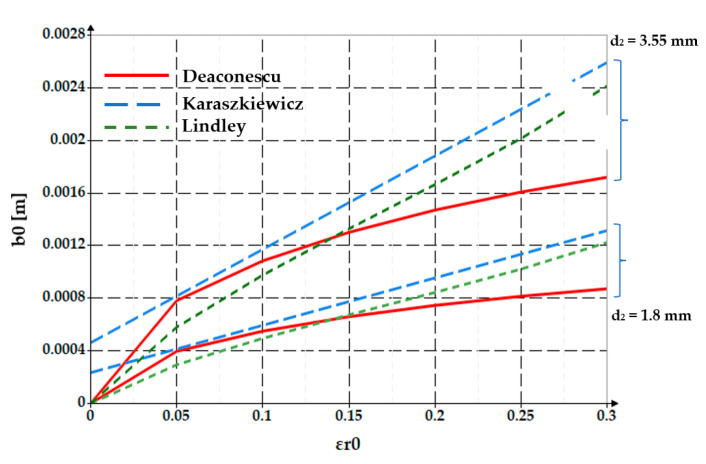
Evolution of the contact area width *b*_0_ versus specific deformation *ε_r_*_0_ and diameter *d*_2_ (2D graph).

Figure 5 shows comparatively the dependencies *b_0_ = f*(*ε_r_*_0_) obtained by Equations (2), (3) and (7). Two values of O-ring diameters were considered, namely *d*_2_ = 1.8 mm and *d*_2_ = 3.55 mm.

**Figure 6:**
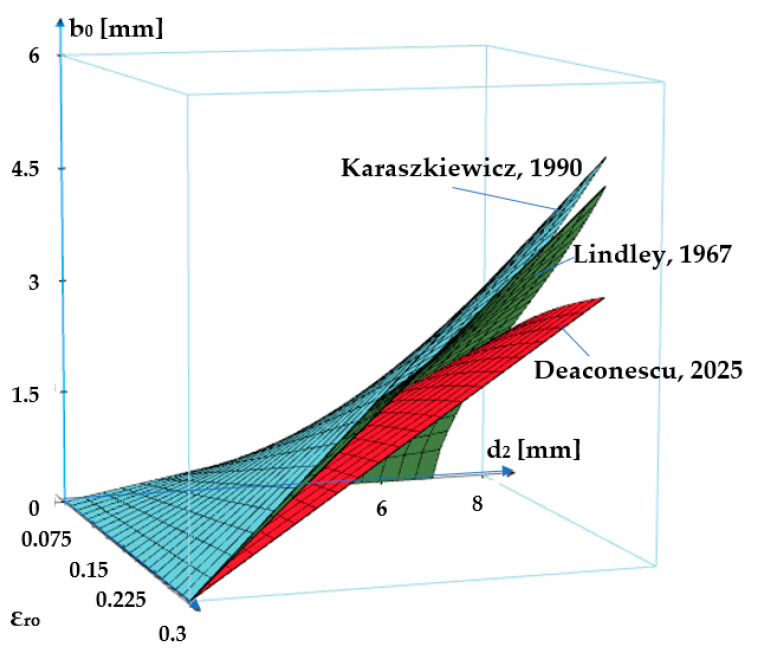
Evolution of the contact area width *b*_0_ versus the specific deformation *ε_r_*_0_ and diameter *d*_2_ (3D graph) [24,26].

The same dependency is shown as a 3D graph in Figure 6.

The two figures show that as the specific radial deformation increases, the width of the contact area grows. The differences between the three approaches (Lindley, Karaszkiewicz, and the authors of this paper) are not significant in the interval *ε_r_*_0_ = 0… 0.2. Beyond this value of the specific radial deformation, the relationship proposed by the authors provides smaller values of the contact area width.

**Figure 7:**
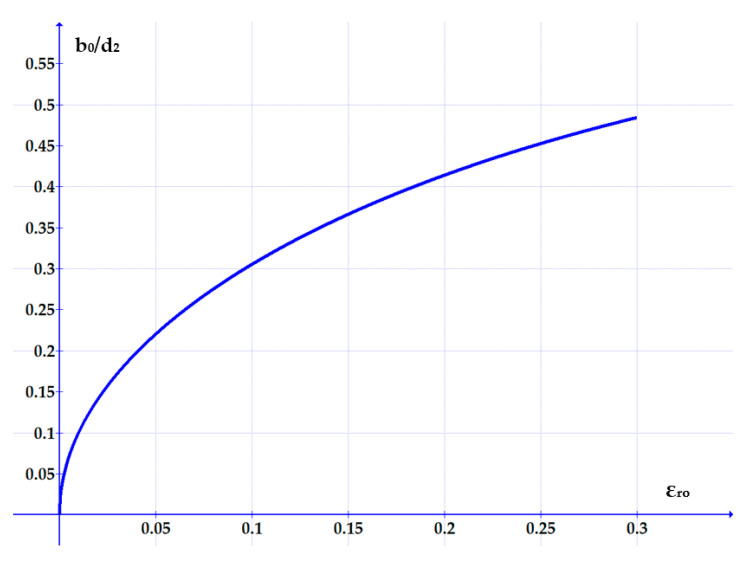
Variation of the non-dimensional ratio *b*_0_/*d*_2_ versus the specific radial deformation.

Starting from Equation (7), the dependency *b*_0_*/d*_2_ *= f*(*ε_r_*_0_) can also be plotted (Figure 7).

**Figure 8:**
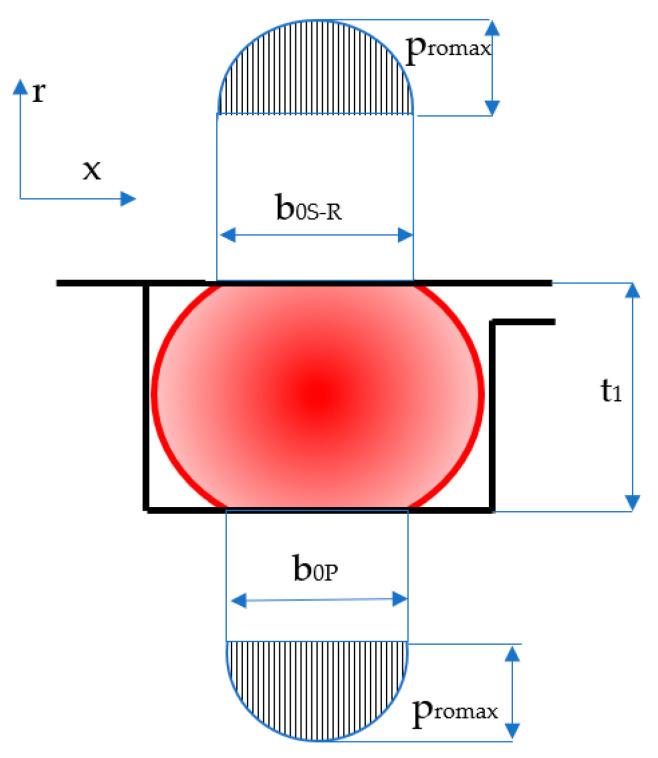
Distribution of contact pressures for zero fluid pressure.

Over the entire width *b*_0_, contact pressures are generated at the interface of the O-ring and its adjacent surfaces, as shown in Figure 8. The width of the contact between the O-ring and the seal was denoted by *b*_0*S-R*_, while *b*_0*P*_ is the width of the contact between the O-ring and the piston of the hydraulic cylinder.

The maximum value of the contact pressures (*p_r_*_0*max*_) was determined starting from Hertz’ theory, resulting in Equation (8):

(8) $$p_{romax}=\frac{\frac{b_{0}}{d_{2}}\cdot E}{4\cdot \left(1-\nu^{2}\right)}$$

where *E* is the equivalent elasticity modulus for the materials of the two contacting bodies, calculated by Equation (9):

(9) $$E=2\cdot \frac{E_{e}\cdot E_{S}}{E_{e}+E_{S}}$$

where *E_e_* is the elasticity modulus of the O-ring material (elastomer), and *E_S_* is the elasticity modulus of the material in contact with the O-ring. In the case of coaxial sealing systems, the O-ring comes into contact with the piston body, on one hand, and on the other, with the seal. For the contact of the O-ring with the piston body (generally made from steel), *E_e_ << E_S_,* so that Equation (9) can be simplified to:

(10) $$E=2\cdot E_{e}$$

In case of the contact between O-ring and seal (generally made from PTFE), there is also a great difference between the values of the elasticity moduli of the contacting materials. Hence, Equation (10) is applicable in this situation too. It can thus be asserted that *b*_0*S-R*_ *= b*_0*P*_ = *b*_0_.

In the literature, numerous relationships between *E_e_* and hardness *H* (Shore A) have been put forward. In ref. [27,28], a finite element simulation study leads to a linear relationship between the logarithm of the elastic modulus and the hardness:

(11) $$\log_{10}E_{e}=c_{1}\cdot H-c_{2}$$

With the *c*_1_ and *c*_2_ empirical constants determined from the best fit as *c*_1_ = 0.0235 and *c*_2_ = 0.6403 for 20 < *H* < 80. Thus, it follows that:

(12) $$E_{e}=10^{0.0235\cdot H-0.6403}$$

For the hardness being expressed in Shore A [27,28], the elasticity modulus is obtained in [MPa]. Upon introducing this latter relationship into Equation (8), the maximum contact pressure becomes:

(13) $$p_{romax}=\frac{\sqrt{\varepsilon_{ro}^{2}\cdot \left(\nu^{2}-1\right)+2\cdot \varepsilon_{ro}\left(1-\nu \right)}\cdot 10^{0.0235\cdot H-0.6403}}{2\cdot \left(1-\nu^{2}\right)}$$

**Figure 9:**
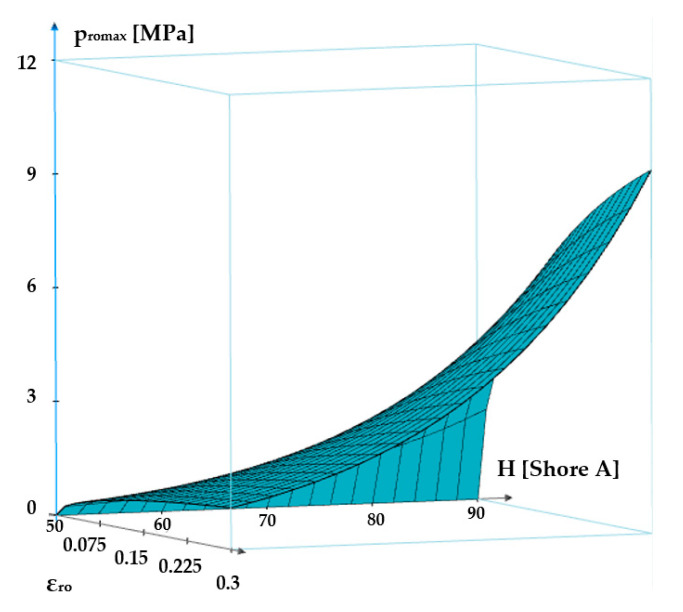
Maximum contact pressure versus specific radial deformation and elastomer hardness.

Figure 9 shows the dependency of the maximum contact pressure on the specific radial deformation and the hardness of the O-ring material.

**Figure 10:**
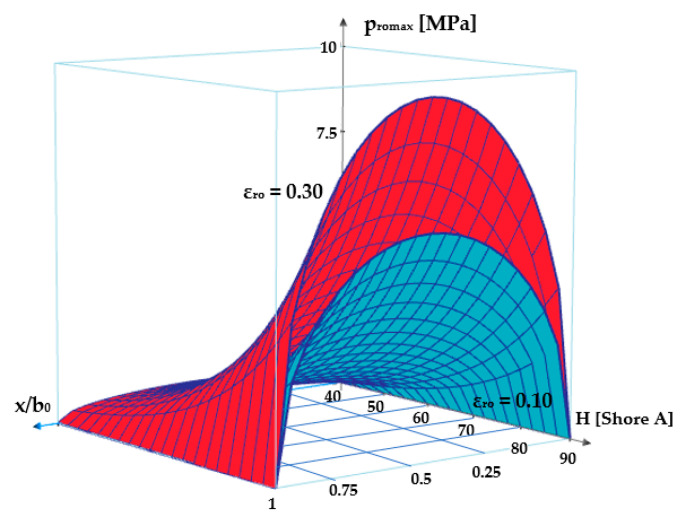
Pressure variation on the width of the contact area.

The distribution of the radial pressure *p_r_*_0_ on the width of the contact area is described by the following parable:

(14) $$p_{ro}=p_{romax}\cdot \sqrt{1-{\left(2\cdot \frac{x}{b_{0}}-1\right)}^{2}}$$

and the dependency *p_r_*_0_ *= f*(*x/b*_0_*, H*) for *ε_r_*_0_ = 0.10 and *ε_r_*_0_ = 0.30 are presented in Figure 10.

It can be noticed that the maximum contact pressure grows with the increase in specific radial deformation and the O-ring’s material hardness.

### 3.2. Behavior of the O-Ring in the Presence of the Fluid Pressure to Be Sealed-Off

**Figure 11:**
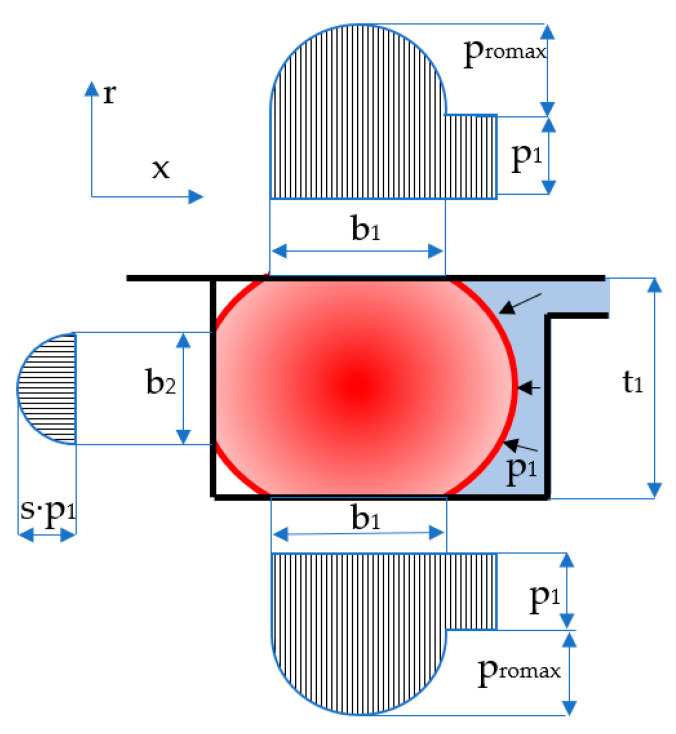
Distribution of the contact pressures for *p*_1_ > 0.

Upon pressure onset in the system, the O-ring will deform additionally, the geometry of its cross-section being modified as shown in Figure 11. Noticeably, three contact areas are generated between the O-ring and the walls of its groove of widths *b*_1_ and *b*_2_.

Experimental research has revealed that due to the viscous component of the O-ring material, the pressure of the working fluid is not transmitted integrally, but is adjusted by a less-than-unity transmission coefficient denoted by *s* that is dependent on Poisson’s coefficient:

(15) $$s=\frac{\nu }{1-\nu }$$

For the studied materials, the considered Poisson’s coefficient was *ν* = 0.496, so that *s* = 0.984.

**Figure 12:**
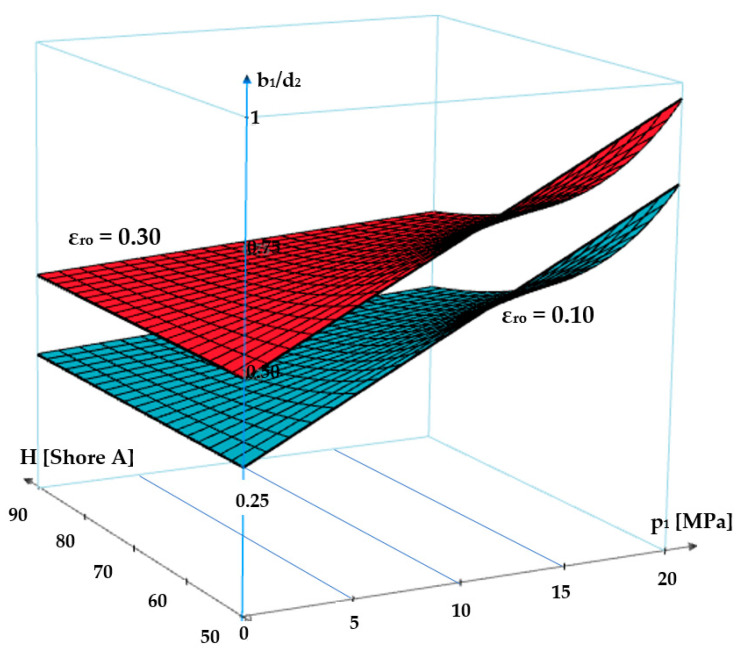
Graph of dependency *b*_1_/*d*_2_ *= f*(*ε_r_*_0_, *p*_1_, *H*).

Starting from Equation (8), the ratio of the contact area width *b_1_* and the diameter of the O-ring cross section (*d*_2_) can be written as:

(16) $$\frac{b_{1}}{d_{2}}=\frac{b_{0}}{d_{2}}+\frac{2\cdot \left(1-\nu^{2}\right)\cdot s\cdot p_{1}\cdot c_{f}}{10^{0.0235\cdot H+0.6403}}$$

where *c_f_* is a coefficient of value 0.083 that describes the modification of shape. The graph in Figure 12 presents the dependency *b*_1_/*d*_2_ *= f*(*ε_r_*_0_, *p*_1_, *H*). It can be noticed that for zero fluid pressure, the width of the contact area is *b*_0_. As the pressure increases, depending on the specific axial deformation and the hardness of the O-ring material, the contact width grows and the value of the *b*_1_/*d*_2_ ratio approaches unity. These findings agree with finite element analyses in large elastic deformations [29] as well as with the experimental measurements [30].

**Figure 13:**
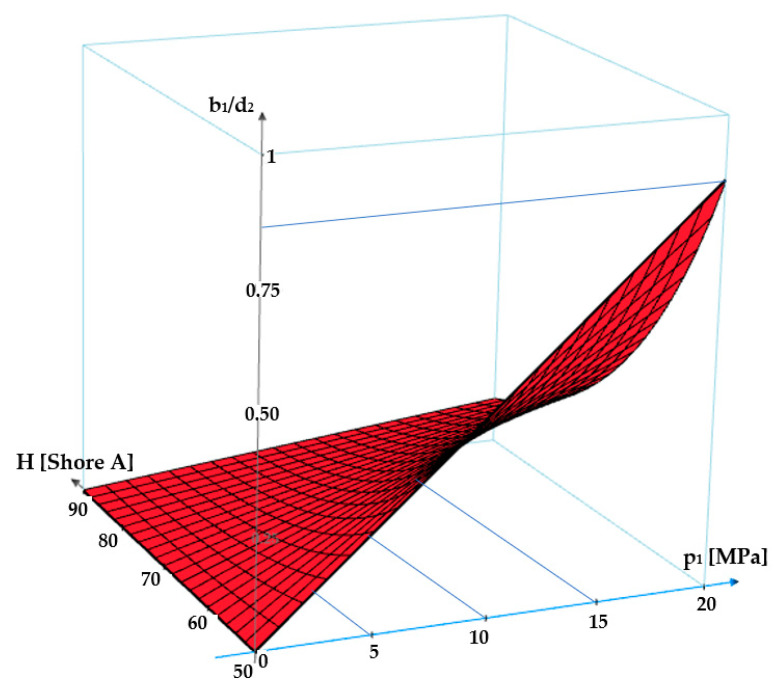
Graph of dependency *b*_2_*/d*_2_ *= f*(*p*_1_, *H*).

A similar calculation yields the ratio *b*_2_*/d*_2_, namely the ratio of the lateral contact area width and the O-ring diameter. Figure 13 shows the dependency *b*_2_*/d*_2_ *= f*(*p*_1_, *H*).

(17) $$\frac{b_{2}}{d_{2}}=\frac{2\cdot \left(s\cdot p_{1}\right)\cdot \left(1-\nu^{2}\right)\cdot c_{f}}{10^{0.0235\cdot H-0.6403}}$$

## 4. Discussion

The graph in Figure 12 shows that an increase in the sealed-off fluid pressure causes greater values of the *b*_1_/*d*_2_ ratio. Furthermore, the less hard the O-ring material is, the greater the ratio *b_1_*/*d*_2_ will be. The specific radial deformation (*ε_r_*_0_) also influences the ratio *b*_1_/*d*_2_, in the sense that a greater value of *ε_r_*_0_ determines a larger width of the contact area.

The variation of the ratio *b*_2_/*d*_2_ is similar (Figure 13). Upon an increase in the pressure of the sealed-off fluid and a decrease in the O-ring material hardness, the ratio *b*_2_/*d*_2_ becomes greater. The specific radial deformation has no influence in this case. The two figures show that *b*_1_/*d*_2_ > *b*_2_/*d*_2_.

In general, it is necessary to know the value of the ratio *b*_2_/*d*_2_ when designing sealing systems with O-rings that work at high pressures. The width of the contact area *b*_2_ exceeding the value *t*_1_ (see Figure 4) causes the extrusion of the O-ring with a negative impact on its durability. In order to avoid extrusion, the use of materials of great hardness (90 Shore A) is recommended. Coaxial sealing systems are not exposed to the risk of extrusion because the length of the lateral contact is restricted by the three walls that support the O-ring (Figure 4).

Regarding the materials used for O-rings, it is recommended to select ones with a hardness of 70 Shore A. The recommended specific radial deformation of the O-ring should fall in a range of 0.18–0.25.

## 5. Conclusions

An optimum selection of materials for seals is an essential requirement for the correct operation of a hydraulic actuation system. This paper analyzes the operational conditions of an O-ring included in a coaxial sealing system, and based on these, recommendations are formulated for the selection of the most adequate materials. A calculation method was developed for the pressures in the contact areas between the O-ring and its adjacent surfaces, and the widths of the contact surfaces were determined. Based on the obtained results, recommendations were issued concerning the optimum characteristics required from polymeric materials in order to be used in coaxial sealing systems. The recommendations offered in this paper are of a general nature and can be used for any materials O-rings are made from. In practice, however, particularly in the case of newly designed sealing systems, further factors may be identified that limit the general applicability of these recommendations. Hence, further research directions will be aimed at identifying and studying such factors, like, but not limited to, the compatibility of coaxial sealing system materials with various working fluids and their behavior at certain unusual temperatures and under various conditions of storage.

## Figures and Tables

**Table 1:** Polymeric materials used for O-rings.

Material	Characteristics
NBR (nitrile butadiene rubber)	low cost, good mechanical performance with resistance to water, most basic oils, lubricants, and some fuels, used in many pneumatic and hydraulic systems
EPDM (ethylene propylene diene monomer rubber)	for applications involving solvents, acids, and other mild chemicals; for water systems, braking systems, medical, pharmaceutical, food, or dairy use
VITON (fluoroelastomer synthetic rubber)	for automotive industry, chemical processing, aerospace, and other application areas with extreme temperature, pressure, and chemical environments
AU (polyester urethane)	good hydraulic oil and gasoline resistance; resistance to mineral and silicone oils and greases; excellent tear and abrasion resistance
FFKM (perfluoroelastomer)	for high temperature, high pressure O-rings, and chemically resistant O-rings

## Data Availability

Data are contained within the article.

## References

[1] Trelleborg Hydraulic Seals. Improving Performance of Hydraulic Cylinders. https://www.trelleborg.com/en/seals/products-and-solutions/hydraulic-seals.

[2] Parker Prädifa Hydraulic Seals. https://www.parker.com/content/dam/Parker-com/Literature/Praedifa/Catalogs/Catalog_HydrSeals_PTD3350-EN.pdf.

[3] Deaconescu T., Deaconescu A. (2014). Film thickness in coaxial sealing systems of hydraulic cylinder rods. J. Balk. Tribol. Assoc..

[4] Deaconescu A., Deaconescu T. (2020). Tribological Behavior of Hydraulic Cylinder Coaxial Sealing Systems Made from PTFE and PTFE Compounds. Polymers.

[5] Krumeich P. (1986). Vom Dichtungselement zum Dichtungssystem. Olhydraul. Pneum..

[6] Wang S., Liu P., Li D., Dong Z., Li G. (2023). Simulation and Experimental Study on Sealing Characteristics of Hydro-Pneumatic Spring GS Seal Rings. Appl. Sci..

[7] Nicolin I., Nicolin B.A. (2019). Physico-mathematical model of the contact between the sealing O-ring and the sealing surfaces. INCAS Bull..

[8] Aissaoui H., Diany M., Azouz J. (2012). Numerical Simulation of Radial and Axial Compressed Elastomeric O-Ring Relaxation. Glob. J. Res. Eng. Mech. Mech. Eng..

[9] Gronau H. (1935). Investigations on Gland Packings and Sealing Rings for High Hydraulic Pressures. Ph.D. Thesis.

[10] Nau B.S. (1999). An historical review of studies of polymeric seals in reciprocating hydraulic systems. Proc. Inst. Mech. Eng. Part J J. Eng. Tribol..

[11] Nau B.S. (1987). The State of the Art of Rubber-Seal Technology. Rubber Chem. Technol..

[12] Sui H., Pohl H., Schomburg U., Upper G., Heine S. (1999). Wear and friction of PTFE seals. Wear.

[13] Weber D., Haas W. (2007). Wear behaviour of PTFE lip seals with different sealing edge designs, experiments and simulation. Seal. Technol..

[14] Sujuan Y., Xingrong Z. (2014). Tribological Properties of PTFE and PTFE Composites at Different Temperatures. Tribol. Trans..

[15] Gül C., Parlar Z., Temiz V. The investigation of frictional characteristics of new design ptfe seals. Proceedings of the 15th International Research/Expert Conference ”Trends in the Development of Machinery and Associated Technology” TMT 2011.

[16] Gong R., Wan X., Zhang X. (2013). Tribological properties and failure analysis of PTFE composites used for seals in the transmission unit. J. Wuhan Univ. Technol.-Mater. Sci. Ed..

[17] Vasilev A.P., Struchkova T.S., Nikiforov L.A., Okhlopkova A.A., Grakovich P.N., Shim E.L., Cho J.H. (2019). Mechanical and Tribological Properties of Polytetrafluoroethylene Composites with Carbon Fiber and Layered Silicate Fillers. Molecules.

[18] Zheng S., Xiao X., Ma X., Li Z., Liu Y., Li J., Wang D., Li X. (2023). Research on Dynamic Sealing Performance of Combined Sealing Structure under Extreme Working Conditions. Appl. Sci..

[19] Kömmling A., Jaunich M., Pourmand P., Wolff D., Hedenqvist M. (2019). Analysis of O-Ring Seal Failure under Static Conditions and Determination of End-of-Lifetime Criterion. Polymers.

[20] Müller M., Mishra R.K., Šleger V., Pexa M., Čedík J. (2024). Elastomer-Based Sealing O-Rings and Their Compatibility with Methanol, Ethanol, and Hydrotreated Vegetable Oil for Fueling Internal Combustion Engines. Materials.

[21] Huang X., Gu J., Li M., Yu X., Liu Y., Xu G. (2023). A Leakage Prediction Model for Sealing Performance Assessment of EPDM O-Rings under Irradiation Conditions. Polymers.

[22] Marco Rubber&Plastics. O-Ring Material Chart—Quick Selection Guide. https://www.marcorubber.com/o-ring-material-quick-reference.htm.

[23] Parker O-Ring Handbook. https://www.parker.com/content/dam/Parker-com/Literature/O-Ring-Division-Literature/ORD-5700.pdf.

[24] Lindley P.B. (1967). Compression characteristics of laterally-unrestrained rubber O-ring. J. IRI.

[25] Lindley P.B. (1966). Load-compression relationships of rubber units. J. Strain Anal. Eng. Des..

[26] Karaszkiewicz A. (1990). Geometry and contact pressure of an O-ring mounted in a seal groove. Ind. Eng. Chem. Res..

[27] Manohar D.M., Chakraborty B.C., Begum S.S. (2012). Hardness–Elastic Modulus Relationship for Nitrile Rubber and Nitrile Rubber–Polyvinyl Chloride Blends. Advances in Design and Thermal Systems.

[28] Qi H.J., Joyce K., Boyce M.C. (2003). Durometer hardness and the stress-strain behavior of elastomeric materials. Rubber Chem. Technol..

[29] George A.F., Strozzi A., Rich J.I. (1987). Stress fields in a compressed unconstrained elastomeric O-ring seal and a comparison of computer predictions with experimental results. Tribal. Znt..

[30] Gorelik B.M., Bukhina B.F., Ratner A.V. (1961). Variation of the contact area in the deformation of rubber cylinders and rings. Sov. Rubber Technol..

